# IL-6 Induced by Periodontal Inflammation Causes Neuroinflammation and Disrupts the Blood–Brain Barrier

**DOI:** 10.3390/brainsci10100679

**Published:** 2020-09-27

**Authors:** Daisuke Furutama, Shinji Matsuda, Yosuke Yamawaki, Saki Hatano, Ai Okanobu, Takumi Memida, Hiroshi Oue, Tsuyoshi Fujita, Kazuhisa Ouhara, Mikihito Kajiya, Noriyoshi Mizuno, Takashi Kanematsu, Kazuhiro Tsuga, Hidemi Kurihara

**Affiliations:** 1 Department of Periodontal Medicine, Graduate School of Biomedical and Health Sciences, Hiroshima University, 1-2-3, Kasumi, Minami-ku, Hiroshima 734-8553, Japan; dftama@hiroshima-u.ac.jp (D.F.); hatanos@hiroshima-u.ac.jp (S.H.); aokanobu@hiroshima-u.ac.jp (A.O.); memida@hiroshima-u.ac.jp (T.M.); tfujita2020@gmail.com (T.F.); kouhara@hiroshima-u.ac.jp (K.O.); mkajiya@hiroshima-u.ac.jp (M.K.); mizuno@hiroshima-u.ac.jp (N.M.); hkuri.hiroshima@gmail.com (H.K.); 2 Department of Advanced Pharmacology, Daiichi University of Pharmacy, 22-1 Tamagawa-cho, Minami-ku Fukuoka 815-8511, Japan; y-yamawaki@daiichi-cps.ac.jp; 3 Department of Advanced Prosthodontics, Graduate School of Biomedical & Health Sciences, Hiroshima University, 1-2-3 Kasumi, Minami-ku, Hiroshima 734-8553, Japan; hiroshi-o@hiroshima-u.ac.jp (H.O.); tsuga@hiroshima-u.ac.jp (K.T.); 4 Laboratory of Cell Biology and Pharmacology, Kyushu University Faculty of Dental Science, 3-1-1 Maidashi, Higashi-ku, Fukuoka 812-8582, Japan; taka-kanematsu@dent.kyushu-u.ac.jp

**Keywords:** periodontal inflammation, neuroinflammation, serum IL-6, BBB permeability

## Abstract

**Background:** Periodontal disease (PD) is a risk factor for systemic diseases, including neurodegenerative diseases. The role of the local and systemic inflammation induced by PD in neuroinflammation currently remains unclear. The present study investigated the involvement of periodontal inflammation in neuroinflammation and blood–brain barrier (BBB) disruption. **Methods:** To induce PD in mice (c57/BL6), a ligature was placed around the second maxillary molar. Periodontal, systemic, and neuroinflammation were assessed based on the inflammatory cytokine mRNA or protein levels using qPCR and ELISA. The BBB permeability was evaluated by the mRNA levels and protein levels of tight junction-related proteins in the hippocampus using qPCR and immunofluorescence. Dextran tracing in the hippocampus was also conducted to examine the role of periodontal inflammation in BBB disruption. **Results:** The TNF-α, IL-1β, and IL-6 levels markedly increased in gingival tissue 1 week after ligation. The IL-6 serum levels were also increased by ligature-induced PD. In the hippocampus, the IL-1β mRNA expression levels were significantly increased by ligature-induced PD through serum IL-6. The ligature-induced PD decreased the claudin 5 expression levels in the hippocampus, and the neutralization of IL-6 restored its levels. The extravascular 3-kDa dextran levels were increased by ligature-induced PD. **Conclusions:** These results suggest that the periodontal inflammation-induced expression of IL-6 is related to neuroinflammation and BBB disruption in the hippocampus, ultimately leading to cognitive impairment. Periodontal therapy may protect against neurodegenerative diseases.

## 1. Introduction

Periodontal disease (PD) has been identified as a risk factor for systemic diseases [1,2,3,4,5]. Recent studies have implicated *Porphyromonas gingivalis* (Pg), a major pathogen of PD, in the pathogenesis of Alzheimer’s disease (AD) [6]. Furthermore, the risk of developing neurodegenerative diseases, including AD, was found to be elevated in patients with PD [7,8]. Sustained neuroinflammation induced by stress or systemic inflammation has been shown to play an important role in the pathogenesis of neurodegenerative diseases, including AD [9,10].

The blood–brain barrier (BBB) comprises blood vessels with endothelial cells that have extremely low rates of paracellular vesicular transport [11] and transcellular vesicular transport (transcytosis) [12,13,14,15,16]. The BBB protects the central nervous system (CNS), which is free of pathogens and toxins under physiological conditions [17]. Compromised BBB functions ultimately result in cognitive impairment, brain damage, and neurodegenerative disorders [12,18,19,20,21,22]. Peripheral inflammation was previously shown to markedly affect CNS functions, leading to cognitive impairment and delirium, due to a compromised BBB [23,24,25,26]. The BBB to macromolecules and most polar solutes is created by tight junctions (TJs) between the cerebral endothelial cells. TJs are a key feature of the BBB and significantly reduce the permeation of polar solutes through paracellular diffusional pathways from the blood plasma to the brain extracellular fluid [27]. The tight junctions consist of proteins spanning the intercellular cleft (occludin and claudins), which are linked to the regulatory proteins Zonula occludens (ZO)-1, ZO-2, ZO-3 [22,28,29].

The relationship between PD and neurodegenerative diseases has been investigated using a PD model administered Pg or bacterial products [30,31]. Although this model is useful for examining the effects of bacterial infections, difficulties have been associated with mimicking sustained periodontal inflammatory responses. In addition, the pathway by which Pg or bacterial products cross the BBB and enter the CNS remains unclear. A ligature-induced PD mouse model is useful for investigating sustained periodontal inflammation in CNS because it exhibits sustained inflammatory cytokine expression in gingival tissue, and the accumulation of bacteria from ligature could not influence systemic conditions [32]. Furthermore, the hippocampus is primarily involved in memory formation. In addition, pathophysiological degeneration in hippocampus is one of the primary hallmarks of AD pathology [33]. Therefore, we examined the role of PD in neuroinflammation in the hippocampus.

Based on these findings, we herein investigated the effects of sustained periodontal inflammation on BBB permeability, a potential mechanism of neuroinflammation in the hippocampus.

## 2. Materials and Methods

### 2.1. Animals and Treatment

Mice were maintained in a vivarium in room temperature and with a 12 h light/dark cycle (lights on at 8:00 AM), and were given ad libitum access to food and water during the experimental period. All the experiments utilizing animals were conducted in accordance with the Guidelines for the Care and Use of Laboratory. The animal experiment procedures were reviewed and approved by the Committee of Research Facilities for Laboratory Animal Science of Hiroshima University (A17-91). Wild-type (WT) C57BL/6j mice (8- to 12-week-old females) were purchased from Charles River Japan (Kanagawa, Japan). Ligature-induced periodontitis in mice was induced according to a previously described method [34]. Briefly, a sterile ligature was placed around the second maxillary molar on both sides in mice until the end of the experiment under anesthesia with butorphanol tartrate (Meiji Seika, Tokyo, Japan), midazolam (SANDOZ, Yamagata, Japan), and medetomidine hydrochloride (Orion, Espoo, Finland). The hippocampus was collected by the following methods. The occipital bone was removed to expose the cerebellum, and then the parietal and frontal bones were immediately removed and the whole brain was collected. The cerebellum and medulla oblongata were dissected out using a scalpel on ice and the cerebrum was divided into left and right hemispheres along the longitudinal fissure. The hippocampal region was collected under a microscope by carefully removing the diencephalon from the cerebrum with forceps. The mouse age was matched at the beginning of the experiment. Mice were decapitated at the end of each experiment. To reveal the role of serum IL-6 in the IL-1β and claudin 5 expression in the hippocampus, recombinant IL-6 (Peprotech, Rocky Hill, NJ, USA, 1000 ng/mouse), IgG control antibody (Leinco Technologies, Fenton, MO, USA, 10 μg/mouse), and anti-mouse IL-6 neutralizing antibody (Bio X Cell, West Lebanon, NH, USA, 10 μg/mouse) were intravenously administered at the experimental schedule.

### 2.2. Real-Time PCR

Previously described methods were used to isolate total RNA from the gingiva and hippocampus [34,35]. The total RNA of gingival tissue and hippocampus was extracted using RNA iso plus (TaKaRa Bio, Siga, Japan). The extraction method was performed according to the manufacturer’s instructions. RNA samples (500 ng) were reverse-transcribed into complementary DNA using ReverTra Ace (Toyobo, Osaka, Japan). Real-time PCR was performed using Light Cycler and SYBR green (Applied Biosystems, Foster City, CA, USA) to assess the relative mRNA expression levels of IL-6, IL-1β, TNF-α, claudin 5, occludin, and ZO1. The PCR thermal profile consisted of initial denaturation at 95 °C for 10 min, followed by 40 cycles at 95 °C for 15 s and 60 °C for 1 min. Fold changes in the genes of interest were calculated by the ∆∆Ct method. The ΔΔCT method directly uses the CT value generated from a qPCR system to calculate the relative gene expression in target and control samples, using GAPDH as the normalizer. ΔCT is the difference between the CT value of a target gene and the CT value of GAPDH. ΔΔCT is the difference between the ΔCT value of a target sample and the ΔCT value of a control sample. The 2^−ΔΔCT^ is the fold change of the target gene expression in a target sample relative to a control sample. The sequences of primers used in the present study are listed in the Supplementary Table.

### 2.3. ELISA

ELISA using the mouse IL-6 Uncoated ELISA Kit (Invitrogen, Carlsbad, CA, USA), mouse IL-1 beta Uncoated ELISA Kit (Invitrogen), and mouse TNF alpha Uncoated ELISA Kit (Invitrogen) was performed using serum in accordance with the instructions of the manufacturers. The limits of detection for each analyte were as follows: mouse IL-6, 4 pg/mL; mouse IL-1β, 8 pg/mL; mouse TNF-α, 8 pg/mL.

### 2.4. Immunofluorescence

Mice were sacrificed and their brains were transcardially perfused with phosphate-buffered saline (PBS). In the immunofluorescence analysis, specimens were embedded in Tissue-Tek OCT compound (Sakura, Torrance, CA, USA), and 12 μm-thick serial sections were prepared in the range including the hippocampus using a cryostat. The BBB disruption was evaluated by claudin 5, ZO-1, and occludin immunostaining. Regarding the claudin 5 staining, brain slices were fixed with methanol for 10 min, rinsed in PBS, and blocked with 5% normal donkey serum (Jackson ImmunoResearch, West Grove, PA, USA). Brain sections were incubated at 4 °C overnight with primary antibodies for CD31 (goat anti-mouse, 1:200, R&D SYSTEM, Minneapolis, MN, USA), claudin 5 (rabbit anti-mouse, 1:100, Thermo Fisher Scientific, Waltham, MA, USA), ZO-1 (rabbit anti-mouse, 1:300, proteintech, Rosemont, IL, USA), and occludin (rabbit anti-mouse, 1:100, proteintech, Rosemont, IL, USA). After rinsing with PBS, brain sections were incubated with the secondary antibodies, donkey anti-rabbit-Alexa 488 (1:100, Invitrogen), and chicken anti-goat-Alexa 594 (1:100, Invitrogen) at room temperature for 2 h. Nuclei were counterstained with 4′,6-diamidino-2-phenylindole (DAPI) (Dojindo, Kumamoto, Japan). Fluorescence signals were detected using an Olympus FV1000D laser scanning confocal microscope (Olympus, Tokyo, Japan). At least five vessels per slide and a total of 10 slides from each animal were randomly acquired. Subsequently, the area of the green fluorescence (claudin 5, ZO-1, occludin) and red fluorescence (CD31) along with blood vessels was measured with the ImageJ software (National Institutes of Health, Bethesda, MD, USA). The quantification of the claudin 5, ZO-1, and occludin protein levels was represented by the ratio of the area expressed for each protein to the area expressed for CD31. The difference in protein level between the target sample and the control sample was evaluated by the ratio of the protein level in the target sample to that in the control sample.

### 2.5. Dextran Imaging

The BBB disruption was evaluated by 3-kDa dextran tetramethylrhodamine lysine fixable labeled with fluorescence (4 mg/mL, Invitrogen), as previously described, with modifications [13]. Each mouse was administered 100 microliters of 3-kDa dextran intravenously. Ten minutes after the administration of dextran, the mice were sacrificed and their brains were transcardially perfused with PBS. The whole brain was harvested. Immunohistochemistry was used to assess BBB disruption as previously described [13]. A 12 μm-thick section was prepared in the range including the hippocampus. Then, 4% PFA was used for the fixation of these sections at room temperature (20–25 °C) for 15 min, then they were rinsed in PBS, blocked with 5% normal donkey serum, and incubated with CD31 for the immunohistochemical imaging of blood vessels. Detections and analysis fluorescence signals were performed in the same way as the immunofluorescence methods. Twenty images (4 slices × 5 images) were counted for each mouse. The green fluorescence along the blood vessels was regarded as the area of BBB disruption. The area of dextran (the green fluorescence) out of the blood vessels was measured with the ImageJ software. The difference in BBB disruption between the ligature mice and the control mice was evaluated by the ratio of the area of green fluorescence in the ligature mice to the area in the control mice.

### 2.6. Statistical Analysis

The Student’s *t*-test was used for the comparisons of two different outcomes from the experiments performed. A one-way ANOVA followed by Dunnett’s test was conducted to assess differences in the inflammatory cytokine expression levels in the gingiva and serum. Outliers, defined as less than the 1st quartile × 1.5 and more than the 4th quartile × 1.5, were excluded from the results (*n* = 1–2). Statistical analysis was performed by JMP^®^ 14 (SAS Institute Inc., Cary, NC, USA).

## 3. Results

### 3.1. Ligature-Induced Gingival and Systemic Inflammatory Responses

**Figure 1:**
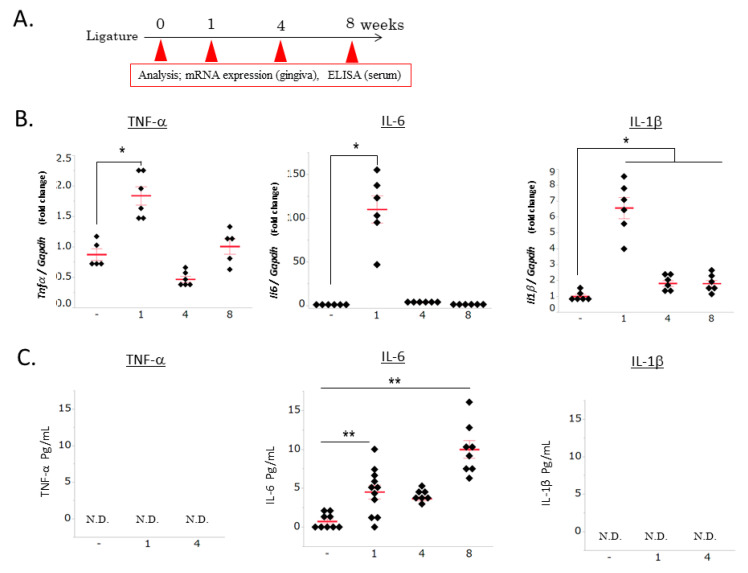
Ligature-induced periodontal inflammation and increased serum IL-6 cytokine levels. (**A**) Schedule of analysis of cytokine mRNA expression levels in the gingiva and protein levels in serum. (**B**) Time course of proinflammatory cytokine mRNA expression in gingival tissue (mean ± SE, *n* = 5–6/group). * *p* < 0.05 significantly different from the control, a one-way ANOVA followed by Dunnett’s test. (**C**) Time course of proinflammatory cytokine protein levels in serum (mean ± SE, *n* = 7–11/group). ** *p* <0.01 significantly different from the control, a one-way ANOVA followed by Dunnett’s test.

To confirm the role of PD in neuroinflammation and BBB disruption, we examined the ligature-induced periodontal inflammation and inflammatory responses in serum. Strong gingival inflammatory responses were observed 1 week after ligation, and then decreased until 8 weeks (*p* < 0.05, Figure 1B). No changes were observed in the TNF-α or IL-1β levels in the serum of mice with ligature-induced PD; however, the IL-6 levels significantly increased 1 week after ligation (*p* < 0.01, Figure 1C). These results indicated this PD mouse model exhibits periodontal inflammation and increased serum levels of IL-6. We determined that ligature placement for 1 week is adequate time to examine the role of periodontal inflammation in pathological change in the hippocampus.

### 3.2. The Role of IL-6 in PD-Induced Increases in IL-1β mRNA Levels in the Hippocampus

**Figure 2:**
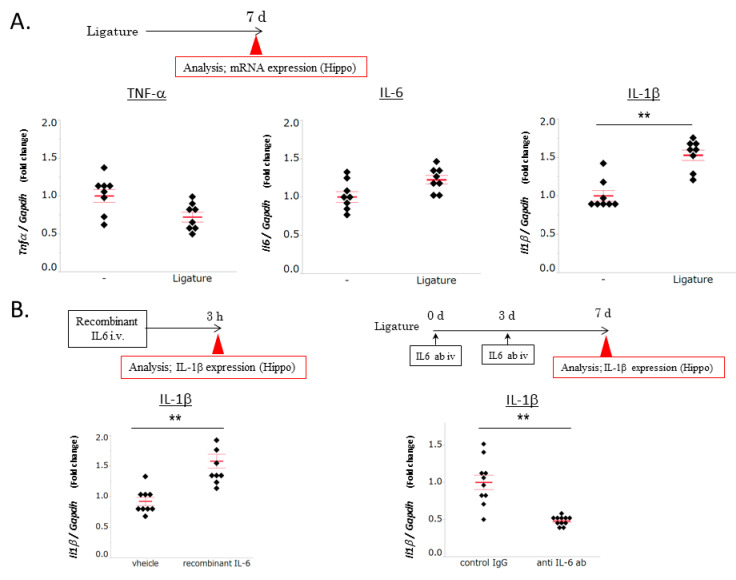
Effects of ligature-induced periodontal disease (PD) on the proinflammatory cytokine expression levels in the hippocampus. (**A**) TNF-α, IL-6, and IL-1β mRNA expression levels in the hippocampus. Ligatures were placed for 1 week (mean ± SE, *n* = 8/group). ** *p* < 0.01 significantly different from the control, the Student’s *t-*test. (**B**) Recombinant IL-6 or vehicle was administered intravenously for 3 h, and the hippocampus was then collected. An anti-IL-6 antibody or control IgG was intravenously injected into ligature-induced PD mice. Antibodies were administered at the beginning and 3 days after ligation (mean ± SE, *n* = 9–11/group). ** *p* < 0.01 significantly different from the control, the Student’s *t-*test.

To examine the effects of PD on inflammatory responses in the hippocampus, the IL-1β, TNF-α, and IL-6 expression levels were measured in ligature-induced PD mice and control mice. The expression levels of TNF-α and IL-6 in the hippocampus remained unchanged by ligation, whereas those of IL-1β significantly increased (*p* < 0.01, Figure 2A). PD increased the serum IL-6 levels in mice. To elucidate the direct role of serum IL-6 in the IL-1β levels in the hippocampus, recombinant IL-6 was intravenously administered to mice without ligation, while a neutralizing antibody for IL-6 was intravenously administered to ligature-induced PD mice to learn about the role of IL-6 upregulation by ligature in serum. The administration of recombinant IL-6 increased the IL-1β mRNA expression levels in the hippocampus (*p* < 0.01), whereas that of the IL-6 neutralizing antibody decreased these levels (*p* < 0.01, Figure 2B). These results indicated that PD-induced increases in the serum IL-6 levels caused an inflammatory response in the hippocampus.

### 3.3. The Role of Ligature-Induced Periodontitis in BBB Disruption

**Figure 3:**
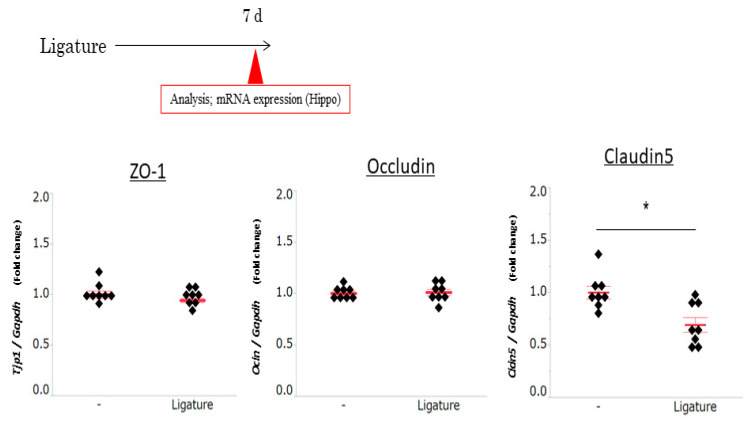
Effects of ligature-induced PD on tight junction-related protein levels in the hippocampus. One week after ligation, the hippocampus was collected and the expression levels of occludin, ZO-1, and claudin 5 were measured (mean ± SE, *n* = 8/group). * *p* < 0.05 significantly different from the control, the Student’s *t-*test.

**Figure 4:**
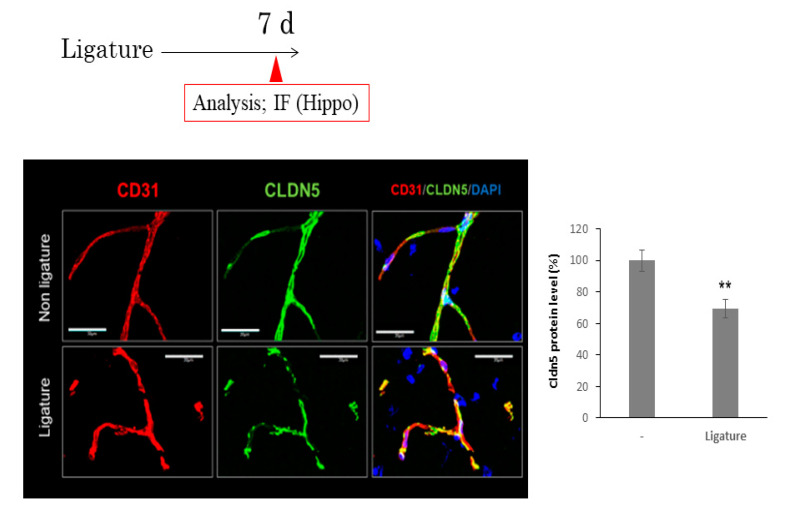
The effect of ligature-induced PD on the levels of claudin 5 on blood vessels in the hippocampus. One week after ligation, the hippocampus was collected. Representative image of claudin 5 (green) is shown along the vessel labeled with endothelial marker (CD31). Blue shows nuclear. Bar = 30 μm. Bar graph shows the ratio area of claudin 5 expression in the vessel. (mean ± SE, *n* = 10/group). ** *p* < 0.01. Significantly different from the control, Student’s *t-*test.

**Figure 5:**
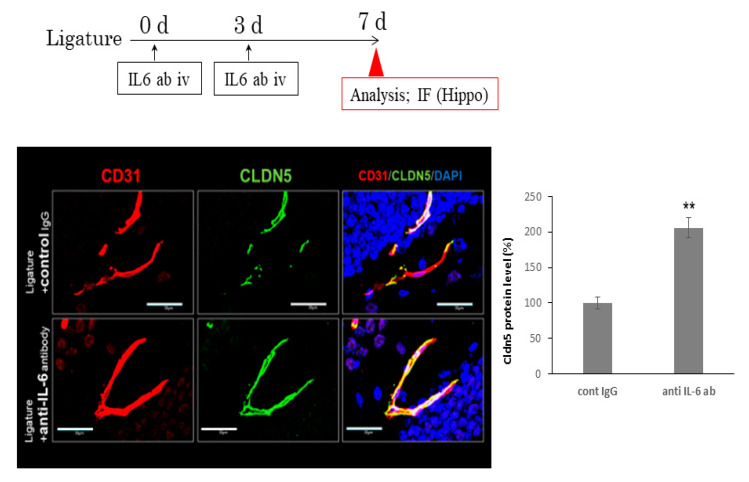
IL-6 antibody prevented the decrease in the levels of claudin 5 induced by PD. Application of IL-6 antibody schedule is shown in scheme. Representative image of claudin 5 (green) is shown along the vessel labeled with endothelial marker (CD31). Blue shows nuclear. Bar = 30 μm. Bar graph shows the ratio area of the claudin 5 expression in the vessel (mean ± SE, *n* = 10/group). ** *p* < 0.01, significantly different from the control, Student’s *t-*test.

**Figure 6:**
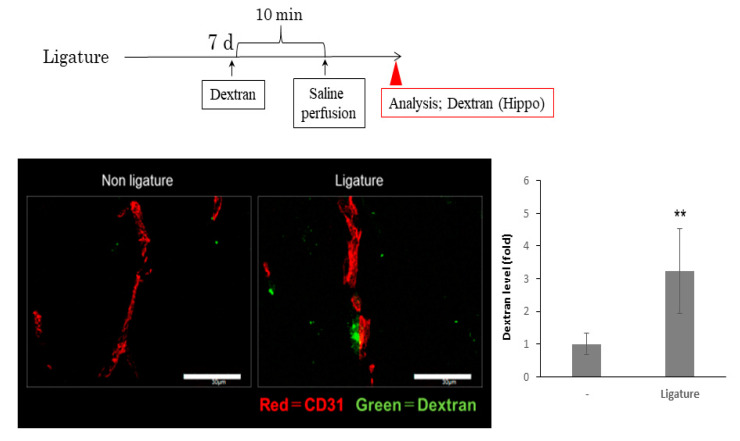
Ligature-induced PD increases the blood brain barrier permeability of dextran in the hippocampus. Ligature was placed for 1 week and then 3-kDa dextran was injected intravenously 10 min before saline perfusion. Representative image of extravascular dextran (green) is shown. The vessel was labeled with CD31. Bar = 30 μm. Bar graph shows the ratio area of extravascular dextran (mean ± SE, *n* = 18–20/group). ** *p* < 0.01, significantly different from the control, Student’s *t-*test.

Since BBB disruption causes neuroinflammation, we investigated the effects of ligature-induced PD on the mRNA expression of tight junction-related proteins. The expression of ZO1 and occludin was not affected by PD. However, ligature-induced PD decreased the mRNA expression levels of claudin 5 in the hippocampus (*p* < 0.05, Figure 3). Consistent with this result, the protein level of ZO1 and occludin was not altered (Figures S1 and S2), although claudin 5 was decreased and the gap area was increased in ligature-induced PD mice (*p* < 0.01, Figure 4). Furthermore, the administration of the IL-6 neutralizing antibody prevented decreases in claudin 5 levels, and the increase in gap area was decreased (*p* < 0.01, Figure 5). Finally, we performed immunohistochemistry on 3-kDa dextran to clarify whether PD increased the BBB permeability. The exhibition of the dextran (green fluorescence) out of the blood vessels (red fluorescence) indicated that it infiltrated brain tissue through the BBB. This can demonstrate the increase in the permeability of the BBB. 3-kDa dextran levels in the hippocampus were higher in the ligature-induced PD mice than in the control mice (*p* < 0.01, Figure 6). However, a bigger molecule, IgG, was not detected in the brain tissue both in the control and ligature-induced PD mice (Figure S3). These results suggested that PD decreased the claudin 5 levels through serum IL-6, thereby increasing the BBB permeability for small molecules in the hippocampus.

## 4. Discussion

In the present study, we reported for the first time that periodontal inflammation increased the IL-1β expression levels in the hippocampus and decreased the claudin 5 levels, which resulted in an increased BBB permeability.

We demonstrated that ligation increased the inflammatory cytokine levels in periodontal tissue and the IL-6 levels in serum. Consistent with previous findings [36,37], we detected changes in the IL-6 levels, but not in the IL-1β or TNF-α levels, in the serum of ligature-induced PD mice. IL-6 is produced by many types of cells and stimulates inflammatory responses [38]. Previous studies have reported that the serum IL-6 levels were higher in PD patients than in a healthy group [39,40]. Whether PD can upregulate serum cytokine levels has not achieved consensus, although many studies have reported that PD increased the IL-6 serum levels [37,41]. Ma et al. reported that ligature increased the serum IL-6 levels but not those of IL-1β. consistent with our findings [42]. This suggests that the serum cytokine levels in ligature-induced PD is not reflected in periodontal tissue inflammation. Ma et al. discussed that this might be caused by transient bacteremia in ligature-induced PD in mice. PD is a risk factor for systemic diseases, including AD [1,2,3,4,5]. It has also been implicated in the pathogenesis of systemic diseases due to bacterial infection and chronic inflammation. A strong relationship has been reported between periodontal pathogen infections (e.g., Pg) and AD [6,31,43,44]. However, the role of PD-induced inflammation in neuroinflammation has remained unclear. We herein demonstrated that ligature-induced PD, a periodontal inflammation model [33,34], increased the IL-6 levels in serum, and this indicates that it is an appropriate model for clarifying the role of PD-induced inflammation in pathophysiological change in CNS systems.

We confirmed that ligature-induced periodontal inflammation significantly increased the IL-1β expression levels in the hippocampus through serum IL-6. The mRNA expression level upregulated by ligature is a 1.5-fold change compared with the control, consistent with previous data showing that posttraumatic stress disorder increased IL-1β expression approximately 1.5-fold, which is connected with neurodegenerative disorder [45]. Sustained increased levels from periodontal inflammation may cause neurodegenerative diseases such as AD. Many stimuli such as ischemia and chronic inflammation increase the IL-1β expression levels in CNS systems [46,47,48,49]. Microglia, resident macrophage cells in the brain, are one of main sources producing IL-1β in the hippocampus [50,51,52]. Inflammatory cytokines through the BBB can activate an inflammatory response in microglia, and this response can trigger a next inflammatory response [53]. In the present study, we confirmed that the intravenous administration of recombinant IL-6 increased the IL-β expression levels in the hippocampus, while the IL-6 neutralizing antibody exerted the opposite effects. Therefore, PD-induced increases in the serum IL-6 levels may further up-regulate the expression of IL-1β in microglia in hippocampus. This finding indicates PD-increased IL-1β in the hippocampus may trigger a next neuroinflammatory response, resulting in the development of neurodegenerative diseases.

To establish whether periodontal inflammation increases the BBB permeability, we examined the expression of tight junction-related proteins in PD mice. The claudin 5 expression levels were lower in ligature-induced PD mice than in control mice, whereas no changes were observed in the ZO1 or occludin expression levels. A previous study demonstrated that the mRNA expression of tight junction-related proteins varies with responses to the stimulation [54]. Further studies are needed to elucidate the mechanisms by which periodontal inflammation decreases the expression levels of claudin 5. Claudin 5 is a critical component of the BBB, based on previous findings showing that claudin 5 deficient mice die within one day after birth because of their dysfunctional blood vessels [55]. The increased serum IL-6 levels in diabetes mellitus rats were shown to reduce the expression of claudin 5 [9]. Furthermore, an in vitro study revealed that IL-6 decreased the claudin 5 levels in endothelial cells through the JAK/STAT pathway [56,57,58]. Consistent with these findings, the decreased expression levels of claudin 5 in the hippocampus were restored by the IL-6 neutralizing antibody. Therefore, PD-induced increases in serum IL-6 levels appear to contribute to BBB disruption. We also investigated whether ligature-induced PD promoted BBB permeability using 3-kDa dextran, and found that extravascular dextran levels were higher in ligature-induced PD mice than in control mice. To examine whether molecules larger than 3-kDa can leak out of the blood vessels, we examined the presence of IgG in the hippocampus of ligature-induced PD mice. However, the leakage of IgG in the hippocampus was not observed in the ligature-induced PD or control mice. These results suggest that PD increases the BBB permeability for small molecules. A previous study showed that the knockdown of claudin 5 and occludin expression in endothelial cells increased the extravascular levels of 3-kDa dextran, but not 10-kDa dextran, in the AD mouse brain [59]. Furthermore, responses to surgery that elevate the serum IL-6 levels increased the BBB permeability and impaired cognition in a mouse model [60]. Collectively, these findings and the present results show that PD induces BBB disruption and plays a role in the pathogenesis of AD.

In the present study, 8-week-old mice were used to investigate whether periodontal inflammation in the short term in young and middle-aged individuals influences brain pathophysiology. In addition, this ligature model would not precisely mimic chronic human clinical pathogenesis. This model similarly reproduced the symptoms of acute periodontal inflammation in gingival tissue. To elucidate whether persistent periodontitis in older ages is involved in the development of neurodegenerative diseases, including AD, further studies with aged mice should elucidate the involvement. BBB disruption has been implicated in cognitive impairment [19,21]. Behavioral experiments on long-term PD, such as ligation for 8 weeks or longer, will reveal the effects of PD-induced inflammation on cognitive impairment.

## 5. Conclusions

In conclusion, the present study revealed that periodontal inflammation induced IL-1β expression in the hippocampus that was dependent on increases in serum IL-6 levels, which ultimately increased the BBB permeability. Further studies are needed to elucidate the mechanisms underlying the relationship between BBB permeability and periodontal inflammation.

## Supplementary Materials

The following are available online at https://www.mdpi.com/2076-3425/10/10/679/s1: Table S1: Sense primers and antisense primers for RT-PCR. Figure S1: Leakage of IgG in the hippocampus. Figure S2: The effect of ligature-induced PD on the levels of ZO1 in blood vessels in the hippocampus. Figure S3: The effect of ligature-induced PD on the levels of Occludin in blood vessels in the hippocampus.
