# Supplementary material for: IL-6 Induced by Periodontal Inflammation Causes Neuroinflammation and Disrupts the Blood–Brain Barrier
Source: Brain Sci. 2020 Sep 27;10(10):679. doi: 10.3390/brainsci10100679 (PMC7599694; doi:10.3390/brainsci10100679)
Supplement: Supplementary file 1 [file brainsci-10-00679-s001.pdf]

Table S1. Sense primers and antisense primers for RT-PCR

Gapdh	Forward	TGGCCTTCCGTGTTCCTACC
	Reverse	GTGTAGCCCAAGATGCCCTTC
Il6	Forward	GAAATGAGAAAAGAGTTGTGCAATGG
	Reverse	ATATCCAGTTTGGTAGCATCCATCAT
Il1b	Forward	CCTTGTGCAAGTGTCTGAAGC
	Reverse	TCATCTTTTGGGGTCCGTCAAC
Tnfa	Forward	CTATGTCTCAGCCTCTTCTC
	Reverse	CATTTGGGAACTTCTCATCC
Cldn5	Forward	AAGGTGTATGAATCTGTGCTGGC
	Reverse	GTGCTACCCGTGCCTTAACTG
Ocln	Forward	CTCGGTACAGCAGCAATGGTAA
	Reverse	TTGTTGATCTGAAGTGATAGGTGGA
Zo1	Forward	CTAAGCCAGTCCATTCTCAGAGC
	Reverse	CCACAGCATCAGTTTCGGGTT

Materials and methods

1, IgG immunostaining

BBB permeability for bigger molecule was assessed by IgG immunostaining. Brain slices were fixed with methanol for 10 min and were permeabilized with 0.5% Triton-X 100 for 45 minutes. Antigens were activated by Liberate Antibody Binding Solution (Polysciences,Inc., PA Warrington, USA).Endogenous peroxidases were inactivated with 0.3% H_2O_2 in methanol. Brain sections were blocked with diluted normal donkey serum (Jackson ImmunoResearch, West Grove, PA, USA) at 4°C overnight, after rinsing with PBS, were incubated with antibodies, donkey anti-mouse-IgG (1:250 R&D systems) at room temperature for 1h. As chromogenic substrate 3,3'-diaminobenzidine tetrahydrochloride (Dako, Redox, Romania, code K3468) was used and counterstained with hematoxylin.

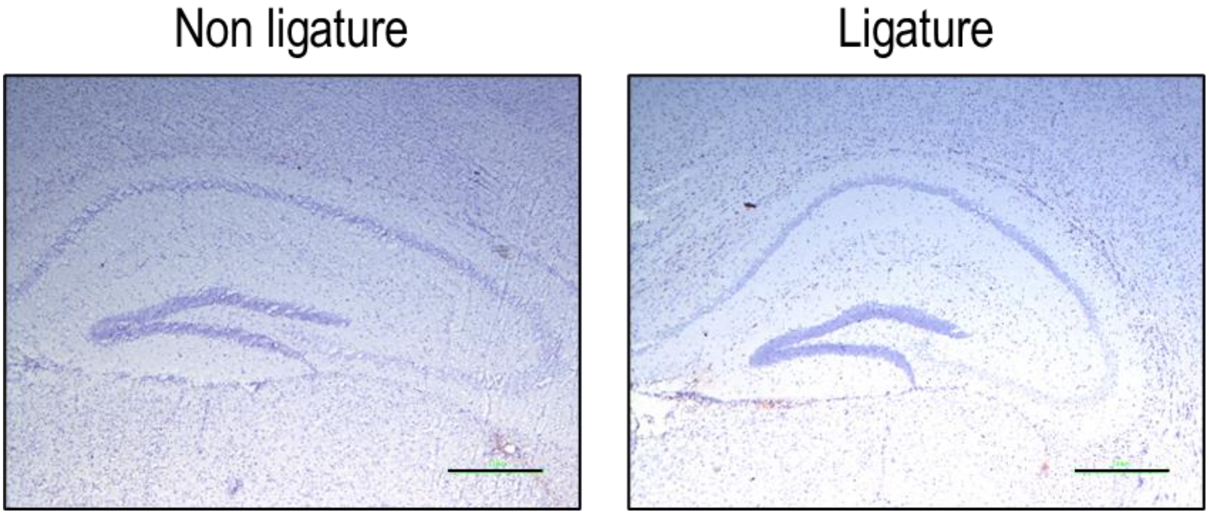

Supplemental figure 1.

Leakage of IgG in hippocampus. IgG were not detected in hippocampus both in non-ligature and ligature mice. Bar=500 μm.

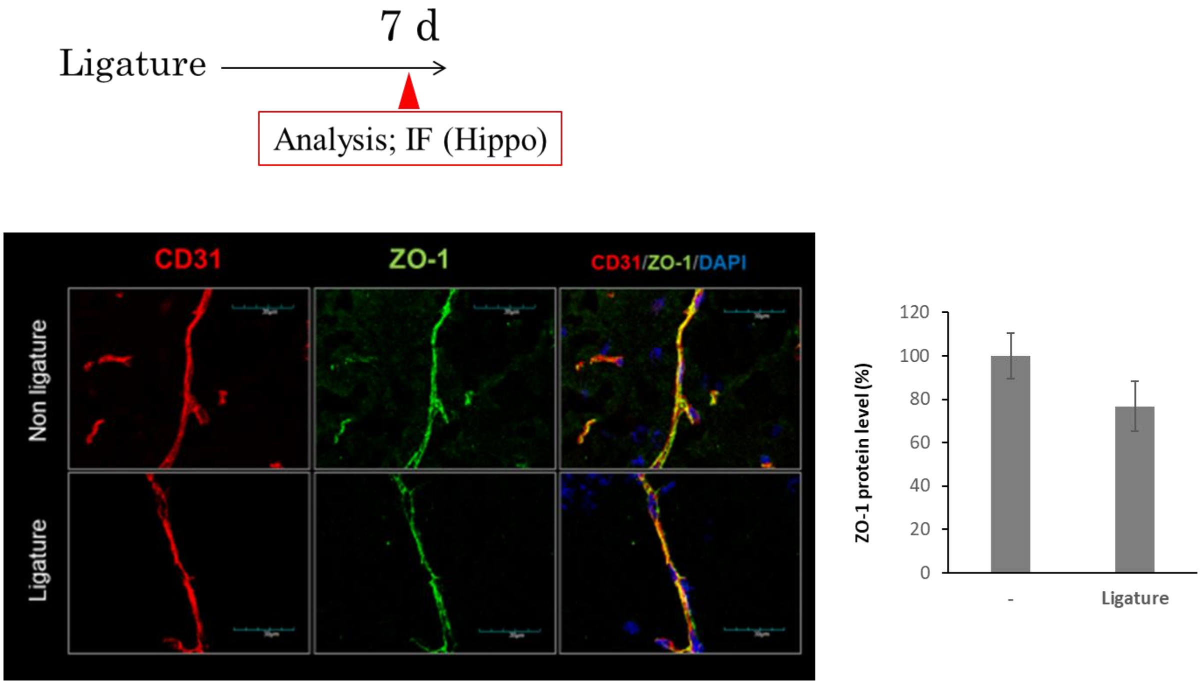

Supplemental figure 2.

The effect of ligature-induced PD in the levels of ZO1 on blood vessel in hippocampus. A. One week after ligation, hippocampus was collected. Representative image of ZO1 (green) was showed along the vessel labeled with endothelial marker (CD31). Blue showed nuclear. Bar=30 µm. Bar graph showed the ratio area of ZO1 expression in the vessel. (mean ± SE, n=10/group).

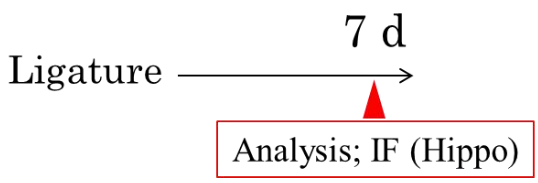

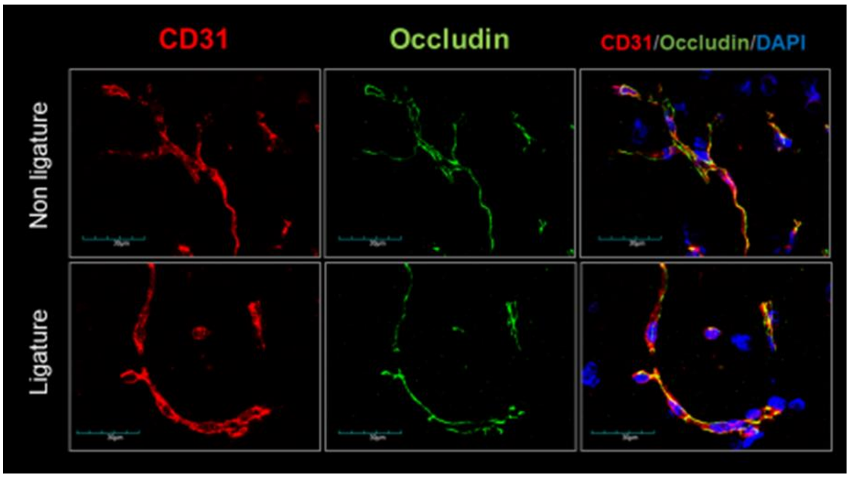

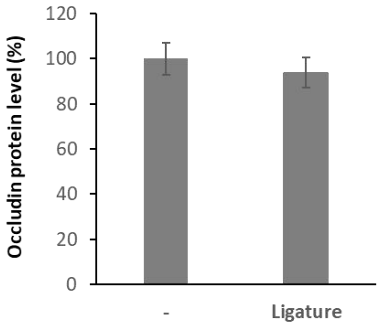

Supplemental figure.3

The effect of ligature-induced PD in the levels of Occludin on blood vessel in hippocampus. A. One week after ligation, hippocampus was collected. Representative image of Occludin (green) was showed along the vessel labeled with endothelial marker (CD31). Blue showed nuclear. Bar=30 µm. Bar graph showed the ratio area of Occludin expression in the vessel. (mean ± SE, n=10/group).
